# Supplementary material for: Dynamic transcriptomic profiles of zebrafish gills in response to zinc supplementation
Source: BMC Genomics. 2010 Oct 11;11:553. doi: 10.1186/1471-2164-11-553 (PMC3091702; doi:10.1186/1471-2164-11-553)
Supplement: Additional file 2 — Interactive Direct Interaction Network representing the molecular interactions between zinc, copper, iron, calcium and proteins encoded by transcripts changed by zinc supplementation. Mini web-site containing index.html and hyperlinked pages in subdirectory describing a Direct Interaction Network automatically generated based on curated interactions contained within the proprietary PathwayArchitect database. Ovals represent proteins and the circles symbolize metal ions. Objects are coloured by their abundance in zebrafish at the time-point they were significantly different from the control is a scale from -4 fold (dark green) to +4 fold (dark red). Where significant differences were found at more than one time-point, the colour overlay shows expression at the first instance. Dark blue squares denote 'binding', and light blue squares 'expression'; green squares stand for 'regulation', green diamonds for 'metabolism', and green circles for 'promoter binding'. Arrow heads indicate directionality of the interaction where annotated. All nodes and edges can be further interrogated by selecting the relative area of the image. [file 1471-2164-11-553-S2.zip › PathwayArchitect Zn xs DIN/465944.html]

# REGULATION:

|  |  |
| --- | --- |
| Type | REGULATION |
| Effect | None |


---

|  |  |
| --- | --- |
| Score | 0 |


---

|  |  |
| --- | --- |
| Reference Count | 6 |


---

|  |  |
| --- | --- |
| Mechanism | Unknown |


---

|  |  |
| --- | --- |
| Reference:0 || Sentence | "We have examined transferrin receptor-1, ferroportin, ceruloplasmin, ferritin light and heavy chains, iron regulatory proteins (IRP)-1 and -2, and hepcidin for mutations that might modulate the iron burden of individuals harboring the common mutant hemochromatosis HFE genotype C282Y/C282Y or cause hemochromatosis independent of mutations in the HFE gene." |
| PMID | 11783942 |
| Year | 2001 |
| Species | Human |
| Journal | Blood Cells Mol Dis |
| RefScore | 0 |
| Source | PArchNLP |
  |
|  |  |


---

|  |  |
| --- | --- |
 Reference:1 || Sentence | "Several new potential mRNA targets for IRP were recently identified: divalent metal transporter-1 (DMT-1) and ferroportin, which are critical regulators of iron absorption in the gut and of iron cycling between various tissues of the body." |
| PMID | 12730455 |
| Year | 2003 |
| Species | Human |
| Journal | J Nutr |
| RefScore | 3 |
| Source | PArchNLP |
  ||  |  |


---

|  |  |
| --- | --- |
 Reference:2 || Sentence | Autosomal dominant iron overload due to a novel mutation of ferroportin1 associated with parenchymal iron loading and cirrhosis. |
| Year | 2004 |
| PMID | 15030991 |
| Species | Human |
| Journal | J Hepatol |
| RefScore | 1 |
| Source | PArchNLP |
  ||  |  |


---

|  |  |
| --- | --- |
 Reference:3 || Sentence | We conclude that, toward the end of the third trimester of iron-sufficient human pregnancy, the placenta accumulates ferritin and potentially increases placental-fetal iron delivery through increased FPN-1 expression. |
| PMID | 15178542 |
| Year | 2004 |
| Species | Human |
| Journal | Am J Physiol Regul Integr Comp Physiol |
| RefScore | 0 |
| Source | PArchNLP |
  ||  |  |


---

|  |  |
| --- | --- |
 Reference:4 || Sentence | IREG1 expression directly correlated with iron content in SH-SY5Y and hippocampal cells. |
| PMID | 15667655 |
| Year | 2005 |
| Species | Mouse |
|  | Human |
|  | Rat |
| Journal | BMC Neurosci |
| RefScore | 1 |
| Source | PArchNLP |
  ||  |  |


---

|  |  |
| --- | --- |
 Reference:5 || Sentence | The genes coding for HFE, transferrin receptor 2 (TFR2), ferroportin (SLC40A1 or FPN1), hepcidin (HEPC) and hemojuvelin (HJV or RGMC) are responsible for different types of genetic iron overload. |
| PMID | 16154838 |
| Year | 2005 |
| Species | Mouse |
| Journal | Haematologica |
| RefScore | 2 |
| Source | PArchNLP |
  |


---

|  |  |
| --- | --- |
